# Tuberculosis in Pregnancy: An Updated Narrative Review

**DOI:** 10.3390/diagnostics16111576

**Published:** 2026-05-22

**Authors:** Carolina Longo, Karina Felippe Monezi Pontes, Marina Matos de Moura Faíco, Mayra Martins Melo, Gustavo Yano Callado, Célio de Barros Barbosa, Edward Araujo Júnior, Antonio Braga

**Affiliations:** 1 Department of Obstetrics, Paulista School of Medicine—Federal University of São Paulo (EPM-UNIFESP), São Paulo 04023-062, SP, Brazil; carolinalongo1989@gmail.com (C.L.); karinamonezi@hotmail.com (K.F.M.P.); mmmoura@gmail.com (M.M.d.M.F.); mayramelo_@hotmail.com (M.M.M.); araujojred@terra.com.br (E.A.J.); 2 Albert Einstein Israelite College of Health Sciences (FICSAE), Albert Einstein Israelite Hospital, São Paulo 05652-900, SP, Brazil; gycallado@gmail.com; 3 Department of Pulmonology, Federal University of Juiz de Fora (UFJF), Juiz de Fora 36038-330, MG, Brazil; celiobbarbosa@gmail.com; 4 Postgraduate Program in Applied Health Sciences, Department of Medicine. Faculty of Medicine, University of Vassouras (Univassouras), Vassouras 27700-000, RJ, Brazil; 5 Discipline of Woman Health, Municipal University of São Caetano do Sul (USCS), São Caetano do Sul 09521-160, SP, Brazil; 6 Department of Gynecology and Obstetrics, School of Medicine, Federal University of Rio de Janeiro (UFRJ), Rio de Janeiro 22240-003, RJ, Brazil; 7 Department of General and Specialized Surgery, School of Medicine and Surgery, Federal University of the State of Rio de Janeiro (UNIRIO), Rio de Janeiro 22290-240, RJ, Brazil

**Keywords:** tuberculosis, pregnancy, diagnosis, chest radiography, computed tomography, maternal outcomes, neonatal tuberculosis

## Abstract

Tuberculosis remains one of the leading infectious causes of morbidity and mortality worldwide, disproportionately affecting women of reproductive age, particularly in low- and middle-income countries. Tuberculosis during pregnancy represents a major clinical challenge, as physiological and immunological changes associated with pregnancy may obscure symptoms, delay diagnosis, and contribute to adverse maternal and perinatal outcomes. This narrative review provides an updated and clinically oriented overview of tuberculosis during pregnancy, with particular emphasis on diagnostic challenges, imaging strategies, microbiological testing, maternal–fetal complications, and therapeutic management. Key topics include symptom-based screening, tuberculin skin test and interferon gamma release assays, as well as molecular diagnostic methods such as GeneXpert Mycobacterium tuberculosis/Rifampicin (MTB/RIF) and Xpert MTB/RIF Ultra, chest radiography, computed tomography, and emerging biomarkers. We also discuss the impact of tuberculosis on pregnancy outcomes, including prematurity, low birth weight, maternal morbidity, and neonatal complications, as well as the particular challenges posed by human immunodeficiency virus HIV coinfection and multidrug-resistant tuberculosis. Current treatment strategies, preventive approaches, postpartum care, neonatal management, and Bacille Calmette–Guérin vaccination are reviewed in light of contemporary evidence and international recommendations. Finally, we highlight practical diagnostic algorithms, current evidence gaps, and priorities for future research aimed at improving maternal and neonatal outcomes in both high- and low-resource settings.

## 1. Introduction

Tuberculosis remains one of the leading infectious causes of mortality worldwide. In 2024, 1.23 million people died from the disease, including 150,000 individuals with human immunodeficiency virus (HIV) coinfection, while an estimated 10.7 million people developed tuberculosis, among them 3.7 million women [1]. Despite this substantial burden, the specific impact of tuberculosis during pregnancy remains insufficiently characterized, as global estimates for pregnant women affected by the disease are not routinely reported by the World Health Organization (WHO) and are absent from official Brazilian statistics [2].

The relevance of tuberculosis in pregnancy is further underscored by its contribution to maternal mortality. In a systematic analysis of causes of maternal death, tuberculosis was included among indirect causes (ICD code O98.0), although not analyzed separately. The study highlighted that indirect causes represent a significant proportion of maternal deaths and are, in most cases, preventable [3].

Beyond maternal health, tuberculosis has important implications for neonatal outcomes. Although congenital tuberculosis is rare, the risk of postnatal transmission is considerable, particularly through inhalation of infectious droplets from maternal cough [4]. Moreover, evidence indicates that maternal and fetal outcomes are significantly worse in women with active tuberculosis, especially when treatment is initiated late, during the second or third trimesters [5].

The development of active tuberculosis is closely associated with social and biological vulnerability. Poverty, malnutrition, harmful use of alcohol and tobacco, and conditions that impair immune function are well-established risk factors [1]. Pregnancy itself has also been proposed as a potential contributing factor, given its immunological adaptations, which may increase susceptibility to disease progression [5,6].

Given the high global burden of tuberculosis, the lack of specific epidemiological data in pregnancy, and the significant maternal and fetal risks associated with the disease, a comprehensive understanding of its epidemiological, pathophysiological, clinical, and therapeutic aspects is essential. Such knowledge is critical to improving early diagnosis and optimizing management strategies, ultimately reducing adverse outcomes for both mother and child.

## 2. Methods

This study was conducted as a narrative review, aiming to provide a comprehensive and integrative synthesis of the current knowledge on tuberculosis during pregnancy, including epidemiology, pathophysiology, clinical presentation, diagnosis, treatment, maternal–fetal outcomes, neonatal management, and public health perspectives.

The choice of a narrative review design was based on the breadth and complexity of the topic, which encompasses heterogeneous domains such as infectious diseases, obstetrics, neonatology, pharmacology, and public health. This approach allows for a contextualized and critical discussion of evidence, integrating data from different study designs and sources, which may not be adequately explored through a strictly systematic methodology alone. A structured literature search was performed in PubMed/MEDLINE, Scopus, and Web of Science. The last search was conducted on 9 May 2026. The search covered publications from January 2000 to May 2026, with older landmark studies included when essential to understanding the historical development, pathophysiology, or clinical evolution of the topic. Additional sources included international guidelines, institutional reports, and technical documents from the World Health Organization (WHO), Centers for Disease Control and Prevention (CDC), and national tuberculosis control programs.

The following search terms and Boolean combinations were used, adapted as appropriate for each database: (‘tuberculosis’ OR ‘Mycobacterium tuberculosis’ OR ‘TB’) AND (‘pregnancy’ OR ‘pregnant women’ OR ‘maternal’ OR ‘postpartum’ OR ‘perinatal’ OR ‘neonatal’ OR ‘congenital tuberculosis’) AND (‘diagnosis’ OR ‘screening’ OR ‘treatment’ OR ‘drug-resistant tuberculosis’ OR ‘multidrug-resistant tuberculosis’ OR ‘MDR-TB’ OR ‘HIV’ OR ‘placenta’ OR ‘breastfeeding’ OR ‘BCG vaccination’ OR ‘prevention’ OR ‘public health’). Additional targeted searches were performed for specific topics, including ‘Xpert MTB/RIF Ultra,’ ‘lipoarabinomannan,’ ‘IP-10,’ ‘tuberculous chorioamnionitis,’ ‘placental tuberculosis,’ and ‘maternal mortality.’

Eligible sources included original observational studies, clinical trials, systematic reviews, meta-analyses, narrative reviews, case series, relevant case reports, guidelines, consensus statements, and official epidemiological reports addressing tuberculosis during pregnancy, postpartum, or the neonatal period. Studies were prioritized when they provided direct evidence on pregnant or postpartum women, maternal and perinatal outcomes, diagnostic performance, treatment safety, drug-resistant tuberculosis, HIV coinfection, placental involvement, or neonatal management. Articles not directly related to tuberculosis in pregnancy or the postpartum period, studies with insufficient clinical relevance, duplicate publications, and sources without accessible full text were excluded. Publications in English, Portuguese, and Spanish were considered.

Priority was given to recent publications and high-quality evidence, while landmark studies were included when essential to understanding the evolution of knowledge in the field. For the purposes of this review, ‘recent’ evidence was defined primarily as literature published within the last 10 years, with particular emphasis on studies, guidelines, and reports published within the last 5 years. ‘High-quality’ evidence was considered to include systematic reviews and meta-analyses, large observational studies, clinical trials, guideline documents, consensus statements, and official reports from recognized national or international health organizations. Landmark studies were included when they were frequently cited, introduced clinically relevant concepts, informed current practice, or provided foundational evidence in areas where recent data remain limited.

Titles and abstracts were screened for relevance by the authors, followed by full-text assessment of potentially eligible articles. The final selection was based on clinical relevance, methodological quality, recency, contribution to the conceptual structure of the review, and ability to support key recommendations. Reference lists of selected articles were also reviewed to identify additional relevant sources.

**Figure 1:**
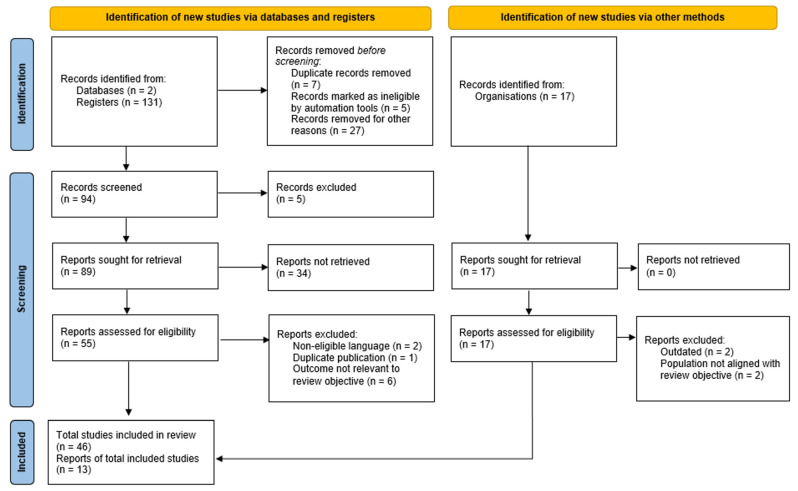
Flow diagram illustrating the literature search and source selection process used in this narrative review. The diagram summarizes the identification, screening, eligibility assessment, and inclusion of studies retrieved from electronic databases, registries, and additional sources relevant to tuberculosis during pregnancy, following PRISMA-informed organizational principles.

Although this article remains a narrative review and was not designed as a formal systematic or scoping review, an adapted flow diagram based on PRISMA principles was included to improve transparency regarding the literature identification and selection process (Figure 1) [7]. No formal risk-of-bias assessment or quantitative synthesis was performed.

As a narrative review, this study is inherently subject to certain limitations, including the potential risk of selection bias in the identification and interpretation of the literature. Although the authors sought to include the most relevant, recent, and high-quality evidence from major databases, international guidelines, and institutional reports, the absence of predefined systematic selection criteria and formal quality assessment may have influenced study inclusion and emphasis. Nevertheless, efforts were made to ensure a balanced, comprehensive, evidence-based, and up-to-date interpretation of the available literature.

## 3. Epidemiology

Estimates of the global burden of tuberculosis, in terms of incidence and mortality, are produced annually by the WHO based on data from national surveillance systems (case notifications and death registries), complemented by special studies such as disease prevalence surveys, mortality surveys, and inventory studies assessing underreporting of diagnosed cases. These estimates are further refined through in-depth analyses of surveillance data and consultation with member states [8].

In 2024, an estimated 10.7 million people developed tuberculosis and 1.23 million died from the disease. The global incidence rate was 131 cases per 100,000 population per year, with a case fatality rate of 11.5%. Tuberculosis remains among the top ten causes of death worldwide and continues to be the leading cause of death from a single infectious agent [9].

The 2025 WHO report included data from 184 countries (out of 215), representing more than 99% of the global population and tuberculosis cases [9]. The burden of disease is highly concentrated, with 30 high-burden countries accounting for 87% of all cases in 2024. Eight countries alone represented 67% of the global total: India (25%), Indonesia (10%), the Philippines (6.8%), China (6.5%), Pakistan (6.3%), Nigeria (4.8%), the Democratic Republic of the Congo (3.9%), and Bangladesh (3.6%) [9].

In 2024, 54% of incident cases occurred in men, 35% in women, and 11% in children. Notably, the absolute number of tuberculosis cases declined for the first time since 2020, following three consecutive years of increase (2021–2023) associated with disruptions in diagnosis and treatment during the COVID-19 pandemic. Although the reduction was modest (1%), from 10.8 million cases in 2023 to 10.7 million in 2024, incidence levels remain above those observed in 2020 (10.3 million) [9].

Understanding the burden of tuberculosis in the general population, and particularly among pregnant women, requires not only an assessment of its epidemiological distribution but also an appreciation of the underlying pathophysiological mechanisms of *Mycobacterium tuberculosis* infection, especially in the context of the immunological adaptations of pregnancy.

## 4. Pathophysiology

*Mycobacterium tuberculosis* (MTB) belongs to a complex of closely related mycobacterial species collectively referred to as the *M. tuberculosis* complex (MTBC), which includes *M. tuberculosis*, *M. bovis*, *M. bovis* Bacille Calmette-Guérin (BCG), *M. africanum*, *M. caprae*, and *M. microti*. Additional species, such as *M. orygis*, *M. mungi*, *M. canettii*, and *M. suricattae*, have also been described, although some remain incompletely characterized [10].

In clinical practice, laboratory identification of *M. tuberculosis* generally refers to detection of the MTBC rather than precise species-level differentiation, as most laboratories lack the advanced methodologies required for subspeciation. This distinction has limited therapeutic relevance, since treatment is largely uniform across the complex, with the notable exception of *M. bovis* and *M. bovis* BCG, which are intrinsically resistant to pyrazinamide [10].

Tuberculosis is primarily acquired through inhalation of viable *M. tuberculosis* bacilli, most commonly transmitted via airborne droplets from individuals with active pulmonary or airway disease [10,11]. Following exposure, infection may result in one of three outcomes: early clearance of the organism, establishment of tuberculosis infection that may persist for decades, or progression to active disease [12,13].

The terminology used to describe asymptomatic infection by *Mycobacterium tuberculosis* has evolved in recent years. The traditional term “latent tuberculosis” has been progressively replaced by “tuberculosis infection,” reflecting a more accurate understanding of this condition as a dynamic spectrum characterized by a persistent immune response to mycobacterial antigens without clinically manifest disease. This shift avoids the misconception of complete biological dormancy and acknowledges the continuous risk of progression to active disease.

It is estimated that approximately one-quarter to one-third of the global population is infected with *M. tuberculosis*, underscoring its extraordinary success as a human pathogen and its status as the leading cause of death from a single infectious agent. This success is largely attributable to highly evolved mechanisms that enable the bacillus to evade both innate and adaptive immune responses.

Advances in genomic technologies have provided unprecedented insights into the evolution of MTBC organisms and the molecular strategies underlying immune evasion. Genome-wide transposon mutagenesis and targeted gene disruption studies have identified a broad array of putative virulence factors involved in host–pathogen interactions across different stages of infection. Nevertheless, for many of these factors, their precise biological roles and contributions to disease pathogenesis remain incompletely understood [13].

## 5. Natural History of the Disease

After inhalation of *Mycobacterium tuberculosis* by a previously uninfected individual, three distinct outcomes may occur. The first is resistance or early elimination of the bacillus. Although the mechanisms underlying this resistance are not fully understood, genetic factors are believed to play a role, with polymorphisms and mutations associated with protection against certain infections. For instance, a 32-base pair deletion in the CC-chemokine receptor 5 (CCR5) gene on CD4+ T cells confers relative resistance to R5-tropic HIV-1 and is associated with delayed disease progression, illustrating the potential impact of host genetic variability on susceptibility to infection [10,12].

The second outcome is tuberculosis infection, characterized by a persistent immune response to *Mycobacterium tuberculosis* antigens in the absence of clinically manifest disease. This condition is identified through cell-mediated immune responses, as detected by interferon gamma release assays (IGRA) or the tuberculin skin test (TST). The third outcome is active tuberculosis, in which clinical manifestations of disease are present and *M. tuberculosis* can be detected in clinical specimens through microscopy, culture, or molecular diagnostic methods [10].

## 6. Risk Factors for Tuberculosis

Certain conditions are consistently associated with an increased risk of progression from tuberculosis infection to active disease. Among these, metabolic disorders such as diabetes mellitus play a significant role, particularly in the presence of chronic hyperglycemia, which impairs innate and adaptive immune responses, including macrophage function and cytokine signaling. Immunosuppression represents another major determinant, most notably in individuals infected with HIV, in whom the depletion of CD4+ T cells compromises the host’s ability to contain *Mycobacterium tuberculosis*, markedly increasing the risk of reactivation and dissemination [10].

Nutritional status is also a critical factor, as malnutrition weakens host defenses by reducing cellular immunity and altering the inflammatory response, thereby facilitating disease progression. In parallel, lifestyle-related factors such as tobacco use and the harmful consumption of alcohol have been independently associated with increased susceptibility to active tuberculosis. Smoking contributes to structural and functional damage of the respiratory epithelium, impairing mucociliary clearance and local immune responses, while chronic alcohol use is linked to both direct immunosuppressive effects and indirect consequences, including poor nutritional status and increased exposure to high-risk environments [10].

These conditions often coexist, particularly in socioeconomically vulnerable populations, creating a cumulative effect that amplifies the risk of tuberculosis progression. Together, they highlight the complex interplay between biological susceptibility and social determinants of health in shaping the global burden of the disease.

## 7. Clinical Presentation

In most cases, infection with *Mycobacterium tuberculosis* remains asymptomatic and non-contagious, with only a minority of infected individuals progressing to active disease. The risk of progression is higher among infants and young children, as well as in individuals with impaired immune responses [1].

Active tuberculosis develops when the bacilli proliferate and disseminate within the host, leading to tissue damage and clinical manifestations that vary according to the organ involved. Although the lungs are the primary site of infection, extrapulmonary involvement may occur, affecting organs such as the kidneys, central nervous system, and spine [1]. The clinical course is often insidious, with symptoms that may be mild and persist for months, contributing to delayed diagnosis and facilitating ongoing transmission, frequently without the individual’s awareness.

The most common manifestations of pulmonary tuberculosis include a persistent cough, occasionally accompanied by hemoptysis, chest pain, progressive weakness, fatigue, weight loss, fever, and night sweats [1]. These symptoms reflect both local pulmonary involvement and systemic inflammatory response. Notably, some individuals may present with minimal or atypical symptoms, particularly in the early stages of disease, which further complicates recognition and control.

During pregnancy, the clinical presentation of tuberculosis may be especially challenging. Symptoms such as fatigue, mild dyspnea, and weight changes can overlap with physiological adaptations of pregnancy, potentially obscuring the diagnosis. In addition, the nonspecific nature of early manifestations may lead to underrecognition or diagnostic delay. In this context, heightened clinical suspicion and the use of appropriate diagnostic strategies are essential to ensure timely identification and management, thereby reducing adverse maternal and fetal outcomes.

## 8. Diagnosis of Tuberculosis During Pregnancy

The diagnosis of tuberculosis during pregnancy remains particularly challenging, as its symptoms, such as fatigue, weight changes, dyspnea, and night sweats, often overlap with normal physiological adaptations of pregnancy. This overlap may lead to delayed recognition and, consequently, to adverse maternal and perinatal outcomes [5,14].

Evidence from a recent systematic review and meta-analysis, including 87 studies conducted in low- and middle-income countries, confirms that tuberculosis detection during pregnancy is significantly impaired not only by nonspecific symptomatology but also by limited access to diagnostic tools [15]. In this context, clinicians should maintain a high index of suspicion, particularly in women from high-burden settings, those living with human immunodeficiency virus (HIV), or those with a history of prior tuberculosis exposure.

The WHO recommends systematic screening of pregnant women in high-burden settings (≥100 cases per 100,000 inhabitants) at each antenatal visit, using a four-symptom-based tool that includes cough, fever, night sweats, and weight loss or inadequate gestational weight gain [16]. Clinical evaluation should consider both pulmonary and extrapulmonary forms of the disease [17]. Immunological tests, such as the TST and IGRAs, are safe during pregnancy. IGRAs offer higher specificity, particularly in Bacille Calmette-Guérin (BCG)-vaccinated populations, and their positivity has been associated with an increased risk of tuberculosis in the postpartum period [18]. However, the physiological immunological shift toward a predominantly anti-inflammatory Th2 profile during pregnancy may attenuate Th1-mediated interferon gamma responses, potentially affecting IGRA performance. In this context, alternative biomarkers have been investigated to improve the detection of tuberculosis infection during pregnancy. Among these, interferon gamma-induced protein 10 (IP-10), a chemokine induced by interferon gamma signaling, has emerged as a promising candidate. Recent studies have demonstrated that IP-10 levels remain elevated in pregnant women with tuberculosis infection, even in the setting of altered interferon gamma responses, suggesting potential utility as a more robust immunological biomarker during pregnancy [19]. Although further validation studies are required before routine clinical implementation, IP-10-based assays may represent an important future adjunct in the diagnosis of tuberculosis infection in pregnant populations. Nevertheless, both TST and IGRAs indicate infection but do not distinguish between tuberculosis infection and active tuberculosis, thus requiring further diagnostic evaluation [20].

Microbiological confirmation remains essential for definitive diagnosis. The GeneXpert MTB/RIF assay, also known as a cartridge-based nucleic acid amplification test (CBNAAT), is a real-time polymerase chain reaction (PCR) method that simultaneously detects *Mycobacterium tuberculosis* and rifampicin resistance within approximately two hours, with sensitivity exceeding 80% and specificity of 99% compared with culture [20]. It is currently recommended as a first-line diagnostic test in high-burden antenatal settings. Conventional methods, including smear microscopy and culture, are still widely used, although they are limited by lower sensitivity and longer turnaround times, respectively. Recent advances in molecular diagnostics have further improved the detection of tuberculosis in paucibacillary disease, a particularly relevant scenario in pregnancy and HIV coinfection. The Xpert MTB/RIF Ultra assay, an enhanced version of the standard Xpert MTB/RIF platform, demonstrates increased sensitivity due to its lower limit of detection and incorporation of multicopy amplification targets. This improved performance is especially valuable in smear-negative and paucibacillary forms of tuberculosis, which are more frequently observed in pregnant women and individuals living with HIV [21]. However, the higher sensitivity of the Ultra assay may be associated with reduced specificity in patients with previous tuberculosis, due to the detection of residual nonviable bacillary DNA. In addition, lateral flow urine lipoarabinomannan (LF-LAM) assays have emerged as useful point-of-care diagnostic tools in severely immunosuppressed HIV-positive patients. These assays detect mycobacterial lipoarabinomannan antigen in urine and may provide rapid bedside diagnosis, particularly in pregnant women with advanced HIV infection, low CD4 cell counts, or severe systemic illness. Although sensitivity remains limited in patients without advanced immunosuppression, LF-LAM testing may facilitate earlier diagnosis and treatment initiation in high-risk populations with substantial diagnostic challenges [22].

**Figure 2:**
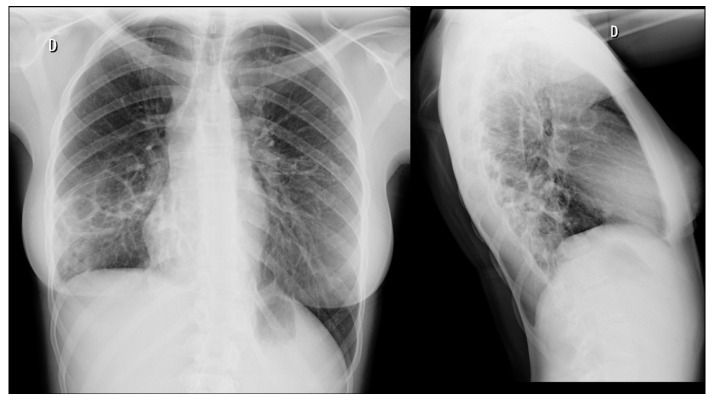
Illustrative chest radiography findings in pulmonary tuberculosis during pregnancy. Posteroanterior and lateral chest radiographs obtained from an anonymized institutional imaging database demonstrate right lung volume loss with ipsilateral mediastinal shift, associated with multiple thin-walled cavitary lesions in the right lower lobe. Additional cavitary involvement is observed in the left upper lobe, illustrating advanced bilateral cavitary pulmonary tuberculosis. The image is included exclusively for educational and radiological illustration purposes within this narrative review.

**Figure 3:**
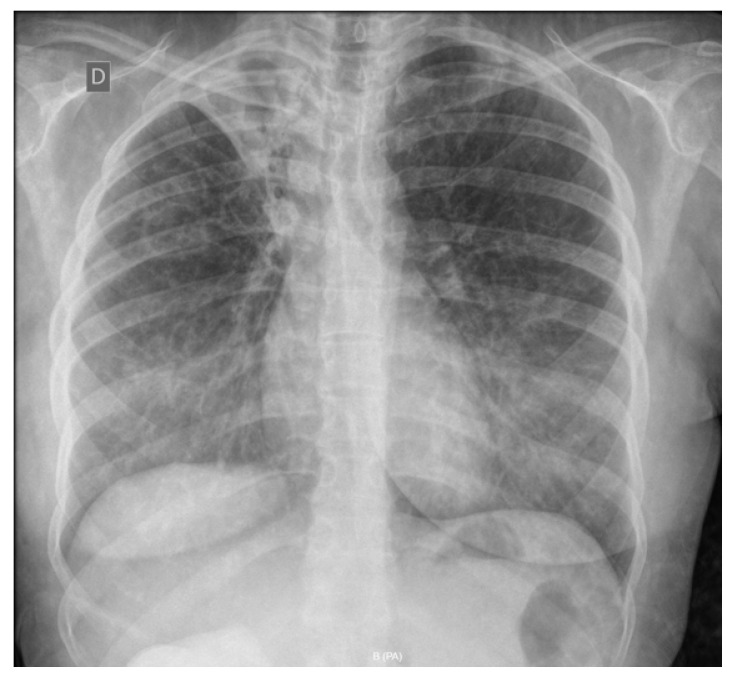
Illustrative chest radiography findings in pulmonary tuberculosis during pregnancy. Posteroanterior chest radiograph obtained from an anonymized institutional imaging database demonstrates right lung volume loss with fibroatelectatic changes involving the right upper lobe. Two cavitary lesions are observed in the upper-mid zone of the right lung, associated with heterogeneous opacities in the left pericardiac region, suggestive of contralateral parenchymal involvement. The image is included exclusively for educational and radiological illustration purposes within this narrative review.

Chest radiography, when performed with appropriate abdominal shielding, is considered safe during pregnancy and provides valuable information regarding the extent and pattern of disease. Its diagnostic benefits clearly outweigh the minimal risks associated with fetal radiation exposure [23]. Radiographic findings in pulmonary tuberculosis may include upper lobe or apical infiltrates, focal or diffuse consolidations, cavitary lesions, hilar or mediastinal lymphadenopathy, pleural effusion, and, in disseminated disease, a classic miliary pattern characterized by diffuse micronodular opacities. Although none of these findings is pathognomonic in isolation, chest radiography remains an essential first-line tool for detecting pulmonary involvement and guiding subsequent investigation. Figure 2 and Figure 3 illustrate representative radiographic findings of pulmonary tuberculosis in pregnancy, highlighting the spectrum of disease presentation, ranging from extensive cavitary destruction with bilateral involvement to asymmetric disease with volume loss, fibroatelectasis, and parenchymal consolidation. These images underscore the variability of radiographic patterns and the potential for advanced pulmonary involvement even in young pregnant patients, including those undergoing treatment or presenting challenges to therapeutic adherence.

Computed tomography (CT) of the chest offers higher diagnostic accuracy than conventional chest radiography for the detection and characterization of pulmonary tuberculosis, particularly in cases with subtle, atypical, or early parenchymal involvement. However, its use during pregnancy must be carefully justified because CT involves higher maternal exposure to ionizing radiation compared with standard chest radiography. Importantly, maternal effective dose and fetal absorbed dose represent distinct dosimetric parameters and should not be interpreted interchangeably. Maternal effective dose reflects the estimated overall biological impact of radiation exposure to maternal tissues, whereas fetal absorbed dose specifically refers to the amount of radiation energy deposited in fetal tissues.

Although chest CT may result in maternal effective doses substantially higher than those associated with conventional chest radiography, fetal absorbed doses from maternal chest CT are considerably lower because the fetus is located outside the primary imaging field and is exposed predominantly to scattered radiation. With contemporary low-dose chest CT protocols, estimated fetal absorbed doses are typically very low and generally remain well below thresholds associated with deterministic fetal effects, including fetal growth restriction, congenital malformations, or neurocognitive impairment. In this context, it is important to distinguish deterministic effects, which occur above specific radiation thresholds, from stochastic effects, for which theoretical risk may increase with cumulative exposure without a defined threshold.

For additional contextualization, the cumulative natural background radiation exposure during an entire pregnancy is estimated to be approximately 0.5–1.0 mSv. This comparison may help contextualize the magnitude of radiation exposure associated with appropriately indicated low-dose chest CT protocols, although background radiation exposure and fetal absorbed dose should not be considered directly equivalent dosimetric measures [24,25].

The use of intravenous contrast in chest CT is generally not recommended in the evaluation of suspected pulmonary tuberculosis during pregnancy. In most cases, non-contrast CT provides sufficient diagnostic information, as findings such as nodules, cavitation, tree-in-bud pattern, and miliary dissemination are adequately visualized without contrast enhancement. Furthermore, iodinated contrast agents cross the placenta and may transiently affect fetal thyroid function. Therefore, contrast-enhanced CT should be reserved for selected situations in which additional diagnostic information is considered essential for maternal management or differential diagnosis [24,25].

**Figure 4:**
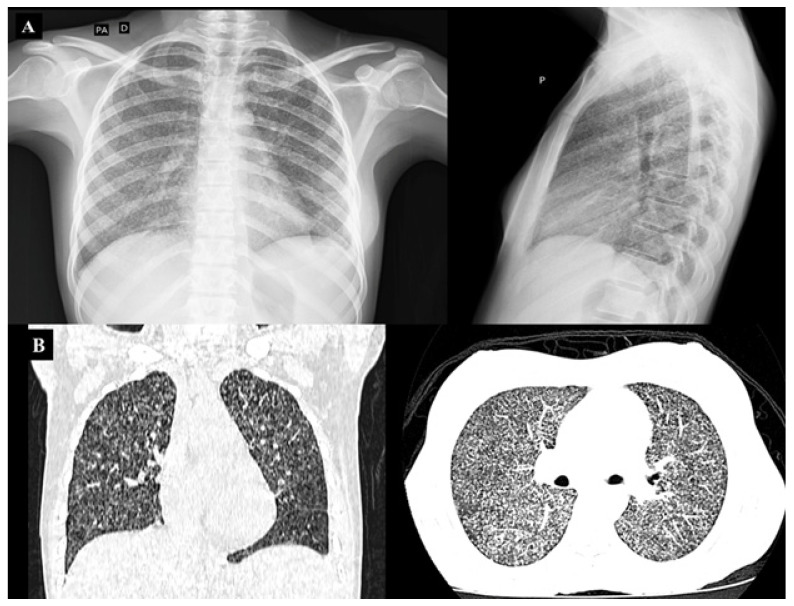
Illustrative imaging findings of miliary tuberculosis during pregnancy. In (**A**), posteroanterior and lateral chest radiographs obtained from an anonymized institutional imaging database demonstrate innumerable diffusely distributed micronodular opacities throughout both lungs, consistent with a classic miliary pattern. In (**B**), coronal and axial maximum intensity projection chest CT reconstructions reveal countless randomly distributed pulmonary micronodules measuring approximately 1–6 mm throughout both lungs, further illustrating the characteristic imaging appearance of miliary tuberculosis. The images are included exclusively for educational and radiological illustration purposes within this narrative review.

**Figure 5:**
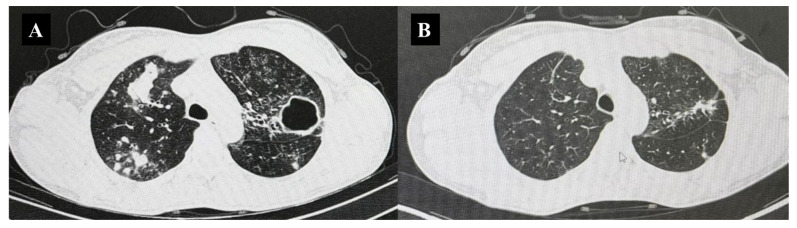
Illustrative chest CT findings of bilateral pulmonary tuberculosis during pregnancy and radiological treatment response. Chest CT images obtained from an anonymized institutional imaging database demonstrate, in (**A**), a thick-walled cavitary lesion in the left upper lobe associated with numerous peripheral bronchiolar opacities exhibiting a tree-in-bud pattern. In the right upper lobe, areas of anterior consolidation with air bronchogram and additional posterior nodular consolidative opacities are observed, illustrating active bilateral pulmonary tuberculosis. Follow-up imaging in (**B**), obtained after completion of treatment with the standard first-line regimen for drug-susceptible tuberculosis during pregnancy, demonstrates marked radiological improvement, including reduction in consolidative opacities, closure of the left upper lobe cavitary lesion with residual linear opacity, and substantial decrease in bronchiolar tree-in-bud opacities, consistent with treatment response. The images are included exclusively for educational and radiological illustration purposes within this narrative review.

Therefore, chest CT should not be considered a routine diagnostic test for suspected tuberculosis during pregnancy, but rather a complementary imaging modality reserved for selected clinical scenarios in which the expected diagnostic benefit outweighs the potential risks associated with radiation exposure. The indication for chest CT in pregnant women with suspected tuberculosis should be individualized and based on careful multidisciplinary risk–benefit assessment. Potential indications include persistent clinical suspicion despite normal or inconclusive chest radiography, particularly in women living with HIV or other immunocompromised states; suspected miliary tuberculosis, in which conventional radiography may have limited sensitivity; severe clinical deterioration or respiratory compromise; evaluation of possible mediastinal or complex parenchymal involvement; differential diagnosis when alternative thoracic conditions are being considered; and selected cases requiring assessment of treatment response when conventional radiographic findings remain inconclusive [24,25,26,27,28,29,30]. Representative cases illustrating the role of chest CT in selected clinical scenarios are shown in Figure 4 and Figure 5. These examples highlight situations in which CT provided critical diagnostic or management information beyond conventional radiography, including suspected miliary tuberculosis with clinical deterioration and complex bilateral pulmonary disease in a socially vulnerable patient with poor treatment adherence.

Although chest CT may be useful in selected situations, chest radiography remains the primary imaging modality in the evaluation of suspected tuberculosis during pregnancy. Its accessibility, safety profile, and diagnostic utility make it the most appropriate first-line tool. Accordingly, the combination of symptom-based screening and chest radiography represents the most effective strategy for case detection in this population. Given the complexity of diagnosis, a multidisciplinary approach involving obstetricians, pulmonologists, and infectious disease specialists is strongly recommended [15]. Despite these strategies, delays in diagnosis remain common, reflecting both clinical and systemic challenges.

The key components of the diagnostic approach to tuberculosis during pregnancy, including their clinical indications and limitations, are summarized in Table 1.

**Figure 6:**
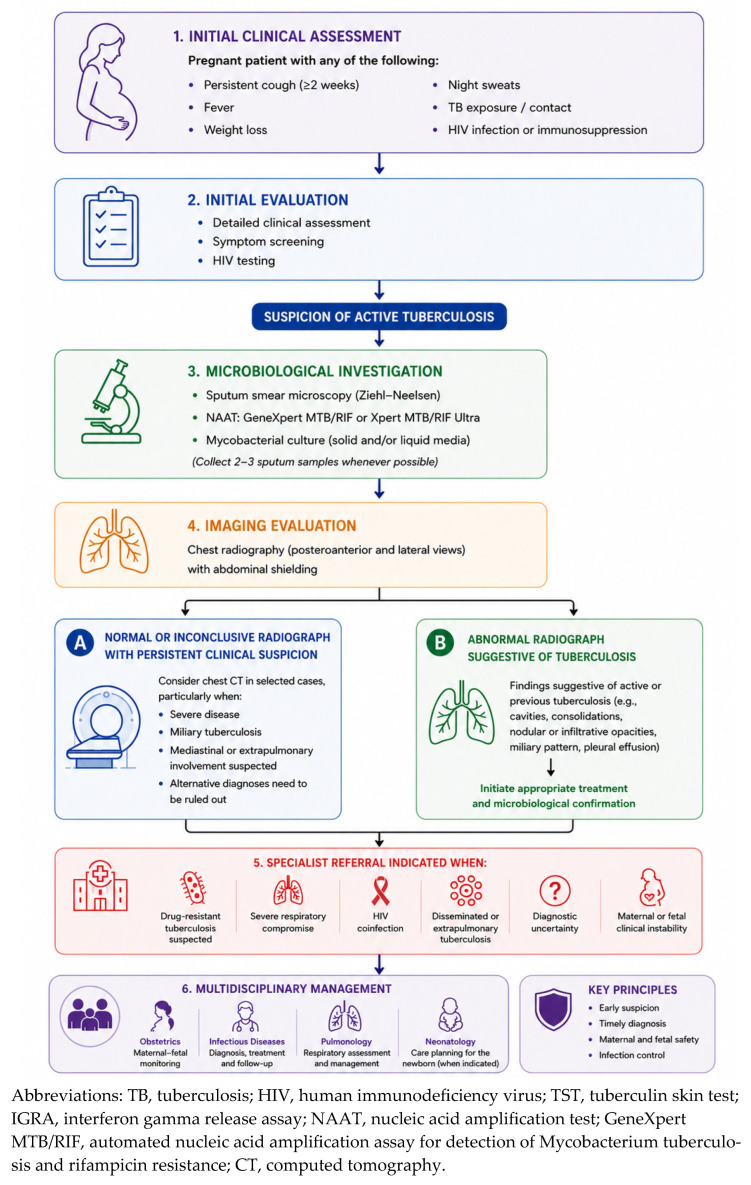
Integrated diagnostic approach for suspected tuberculosis during pregnancy: a practical algorithm incorporating clinical evaluation, microbiological testing, imaging assessment, and specialist referral.

To further facilitate the practical application of these diagnostic principles in clinical settings, Figure 6 presents a proposed diagnostic algorithm for the evaluation of suspected tuberculosis during pregnancy, integrating symptom screening, microbiological investigation, imaging assessment, and indications for specialist referral.

## 9. Impact of Tuberculosis During Pregnancy

Tuberculosis during pregnancy is associated with a broad spectrum of adverse maternal and perinatal outcomes, particularly when diagnosis and treatment are delayed. In a meta-analysis conducted by Sobhy et al. [5], active tuberculosis was consistently linked to unfavorable outcomes in both mothers and infants, underscoring the clinical relevance of timely detection and appropriate management. Although an increased risk of maternal death was observed, this association did not reach statistical significance; notably, among maternal deaths, a substantial proportion of women were coinfected with HIV, highlighting the synergistic impact of immunosuppression on disease severity [31].

The same analysis demonstrated that outcomes were particularly poor when extrapulmonary tuberculosis was diagnosed later in pregnancy, especially during the second and third trimesters. Reported complications included maternal anemia, perinatal death, preterm birth, low birth weight, and fetal distress in women with active disease [5]. These findings emphasize not only the severity of tuberculosis in pregnancy but also the critical importance of early diagnosis.

Pregnancy itself may favor the progression from tuberculosis infection to active disease, particularly in the context of repeated or closely spaced pregnancies, in which cumulative physiological and immunological changes may increase susceptibility to disease activation [32]. Moreover, the overlap between tuberculosis symptoms and normal gestational changes frequently leads to diagnostic delay, which is a key determinant of worse maternal prognosis [5,32].

Overall, tuberculosis during pregnancy is associated with increased risks of prematurity, low birth weight, and heightened perinatal morbidity and mortality. These risks are further amplified in cases of delayed diagnosis or inadequate treatment, leading to more severe clinical complications for both the mother and the fetus.

## 10. Complications in Pregnant Women with Tuberculosis and Their Management

Active tuberculosis during pregnancy is associated with substantial maternal and perinatal morbidity and mortality. A systematic review conducted by Sobhy et al. demonstrated a threefold increase in overall maternal morbidity, a fourfold increase in perinatal mortality (odds ratio 4.2), a ninefold higher rate of miscarriage, and a 1.7-fold increase in preterm birth among affected pregnancies [5]. The occurrence and severity of complications are influenced by multiple factors, including disease burden, drug resistance, gestational age at diagnosis, HIV coinfection, and adherence to treatment [5].

### 10.1. Hypertensive Disorders

Hypertensive disorders appear to be more frequent in pregnant women with tuberculosis. A 2024 meta-analysis including studies from Asia and Africa suggested that systemic inflammation and endothelial dysfunction associated with infection may impair placental vascular development, thereby increasing the risk of hypertensive disease [15].

This association has important therapeutic implications. Rifampicin, a cornerstone antituberculosis drug, induces the cytochrome P450 enzymatic system, accelerating the metabolism of several medications and potentially reducing the efficacy of certain antihypertensive agents, including methyldopa. In this context, beta-blockers or calcium channel blockers are often preferred, with close monitoring to ensure adequate blood pressure control [14].

### 10.2. Chorioamnionitis

Hematogenous dissemination of *Mycobacterium tuberculosis* to the placenta may result in tuberculous chorioamnionitis, particularly in cases of disseminated or genital tuberculosis. Studies have demonstrated a significantly increased risk of chorioamnionitis in pregnant women with tuberculosis at the time of delivery compared with unaffected controls.

This condition is associated with adverse outcomes such as preterm birth, neonatal sepsis, and congenital tuberculosis. When vertical transmission is suspected, histopathological examination of the placenta and mycobacterial cultures at delivery are recommended [20]. Recent clinicopathological studies have demonstrated that placental tuberculosis may present with both classic granulomatous lesions and nonspecific inflammatory findings. Gross examination may reveal placental enlargement, gray–white necrotic foci, focal yellow–white nodules, caseous areas, focal calcifications, or diffuse thickening of the fetal membranes. Microscopically, although epithelioid granulomas with Langhans-type multinucleated giant cells and caseous necrosis may be identified, these classic findings are not universally present. In contrast, acute fetal membranitis and chorioamnionitis with prominent neutrophilic infiltration appear to be more frequent findings, often associated with inflammatory necrosis, coagulative necrosis, and focal involvement of the chorion and amnion. Chronic inflammatory infiltrates involving the decidua and chorionic villi may also be observed. Acid-fast bacilli may occasionally be detected using Ziehl–Neelsen staining, while molecular assays and mycobacterial culture provide important complementary confirmation due to the frequently paucibacillary nature of placental disease [33].

### 10.3. Diabetes Mellitus

Diabetes mellitus (DM) and tuberculosis have a well-established bidirectional relationship. DM impairs host immune responses against *Mycobacterium tuberculosis*, while the inflammatory state associated with tuberculosis may worsen glycemic control. During pregnancy, this interaction becomes even more pronounced [31].

Gestational diabetes has been associated with reduced levels of interferon gamma (IFN-γ), a key mediator of host defense against mycobacterial infection, with greater impairment observed in women coinfected with HIV [18]. In addition, rifampicin-induced activation of cytochrome P450 enzymes may alter glucose metabolism and insulin requirements, necessitating strict glycemic monitoring. At the population level, the increasing prevalence of diabetes has contributed to a growing burden of tuberculosis–diabetes comorbidity, which in some settings now exceeds tuberculosis–HIV coinfection [14].

### 10.4. Postpartum Hemorrhage

The risk of postpartum hemorrhage (PPH) is increased in pregnant women with tuberculosis. This may result from uterine atony in the context of nutritional depletion, as well as from rifampicin-induced alterations in the metabolism of vitamin K-dependent coagulation factors [34].

A recent study confirmed higher rates of anemia, PPH, and pregnancy-related death in women with active tuberculosis. When rifampicin is used near term, prophylactic administration of vitamin K to both the mother and the newborn is recommended [35].

### 10.5. Anemia and Sepsis

Maternal anemia is significantly more frequent in pregnancies complicated by tuberculosis, occurring up to four times more often than in unaffected pregnancies. This is typically multifactorial, resulting from chronic inflammation, nutritional deficiencies, and, in some cases, drug-related hemolysis [32].

Severe infections, including sepsis, may occur in cases of disseminated tuberculosis or secondary bacterial infection, particularly in immunocompromised women. Pregnant women hospitalized with tuberculosis have been shown to have higher rates of acute respiratory distress syndrome and increased need for mechanical ventilation [14].

### 10.6. Rupture of Pulmonary Bullae

Pulmonary bullae, which may develop as sequelae of cavitary tuberculosis, can rupture during pregnancy due to increased intra-abdominal pressure associated with uterine enlargement. This may result in secondary pneumothorax and, in severe cases, tension pneumothorax or acute respiratory failure.

In women with known pulmonary bullae, assisted vaginal delivery, such as vacuum extraction, may be considered to shorten the second stage of labor and reduce intrathoracic pressure associated with the Valsalva maneuver [14].

### 10.7. Tuberculosis and Maternal Mortality/Tuberculosis as a Cause of Maternal Death

Tuberculosis is recognized as one of the leading non-obstetric infectious causes of maternal mortality worldwide, particularly in high-burden settings [13,30,31,32]. A recent modeling study estimated that, in 2023, approximately 239,500 pregnant women and 97,600 postpartum women developed active tuberculosis globally, corresponding to about 9% of all tuberculosis cases among women aged 15 years or older. The highest burden was observed in the WHO Southeast Asia Region, followed by the African Region [36]. Among pregnancy-associated tuberculosis cases, HIV coinfection was present in 21% during pregnancy and 11% in the postpartum period, underscoring the important overlap between these two epidemics [37].

Despite its impact, maternal mortality related to tuberculosis is likely underestimated. Most national tuberculosis control programs do not routinely record pregnancy status in reported cases, and verbal autopsy methods frequently fail to accurately identify tuberculosis as the underlying cause of death [38]. Evidence from sub-Saharan Africa illustrates the magnitude of this gap: in Mozambique, tuberculosis was identified as the cause of death in 12.9% of all maternal deaths and in 27.7% of deaths among HIV-positive women in an autopsy-based study [39]. Similarly, in South Africa, non-obstetric infections, predominantly tuberculosis, accounted for 47% of maternal deaths up to 2007, while in Nigeria, tuberculosis–HIV coinfection represented 17.74% of non-obstetric deaths among women of reproductive age [31].

The postpartum period has been associated with an increased risk of tuberculosis progression and clinical deterioration compared with both pregnancy and non-gestational periods. Large population-based studies have consistently demonstrated that tuberculosis incidence increases after delivery, with rates peaking in the months following childbirth [2]. Among women living with HIV, the coexistence of tuberculosis in the postpartum period is associated with a twofold increase in maternal mortality, while their newborns have a threefold higher risk of death compared with those born to HIV-positive women without tuberculosis [16].

Addressing tuberculosis-related maternal mortality requires coordinated public health strategies. These include early diagnosis and prompt initiation of treatment, strengthening of surveillance systems, such as the mandatory recording of pregnancy status in tuberculosis notification forms in Brazil since 2007, and the integration of systematic tuberculosis screening into antenatal and postpartum care [40].

In this context, coinfection with HIV plays a central role, as it markedly increases the risk of progression from tuberculosis infection to active disease and is associated with more severe clinical outcomes. This interaction demands heightened vigilance and a more comprehensive diagnostic and therapeutic approach throughout pregnancy and the postpartum period.

## 11. Coinfection with Human Immunodeficiency Virus

HIV and tuberculosis constitute synergistic epidemics with particularly severe consequences during pregnancy [34]. HIV infection is the strongest known risk factor for progression from tuberculosis infection to active disease, with an estimated annual risk of approximately 10%, compared with a lifetime risk of 5–10% in immunocompetent individuals [20]. Consistent with this, the prevalence of active tuberculosis during pregnancy is substantially higher among HIV-positive women, ranging from 0.7% to 11%, compared with 0.07% to 0.5% in HIV-negative populations.

Tuberculosis–HIV coinfection is associated with substantially worse maternal and neonatal prognosis and has been linked to increased maternal and infant mortality in high-burden settings [31,38,39]. In addition, tuberculosis increases the risk of vertical transmission of HIV to the fetus, further amplifying neonatal vulnerability [31]. Notably, more than half of pregnant women who die from tuberculosis are HIV-positive, highlighting the critical role of immunosuppression in disease severity [38]. A comprehensive review by Yilma et al. [39] confirmed that tuberculosis–HIV coinfection during pregnancy adversely affects maternal health, pregnancy progression, and neonatal outcomes across multiple domains.

The clinical presentation of tuberculosis in HIV-positive pregnant women is often atypical. Smear-negative disease, extrapulmonary involvement, and normal or nonspecific chest radiographic findings are more common, making diagnosis more challenging and reinforcing the need for a high index of suspicion and comprehensive microbiological evaluation [41].

Management is further complicated by pharmacological interactions between antituberculosis drugs and antiretroviral therapy. Rifampicin, a key component of standard tuberculosis treatment, induces the cytochrome P450 (CYP3A4) enzymatic system, significantly reducing plasma concentrations of several antiretroviral agents, particularly nevirapine [31]. Efavirenz is generally preferred due to its more favorable interaction profile, although its use in early pregnancy requires careful consideration. Alternatively, rifabutin may be used in place of rifampicin to mitigate these interactions [41].

The timing of treatment initiation is also critical. Antiretroviral therapy should typically be initiated within two to eight weeks after the start of antituberculosis treatment, balancing the benefits of early viral suppression with the risk of complications [37]. In addition, the World Health Organization recommends that all HIV-positive pregnant women without active tuberculosis receive tuberculosis preventive therapy, with isoniazid administered for at least 36 months in high-transmission settings [42].

Clinicians should also be aware of immune reconstitution inflammatory syndrome, a condition characterized by paradoxical worsening of tuberculosis following the initiation of antiretroviral therapy. This phenomenon may occur during pregnancy or in the postpartum period and requires continuation of both antituberculosis and antiretroviral treatments, with careful clinical monitoring [31].

## 12. Treatment of Tuberculosis During Pregnancy

Given the significant impact of tuberculosis during pregnancy, timely and appropriate treatment represents the cornerstone for reducing maternal and fetal morbidity and mortality. Management requires careful consideration of drug safety, adherence, and close clinical follow-up.

Treatment of active tuberculosis during pregnancy should not be delayed, as the risks associated with untreated disease for both the mother and the fetus clearly outweigh those related to pharmacological therapy [14]. The standard first-line regimen for drug-susceptible tuberculosis—2HRZE/4HR (isoniazid, rifampicin, pyrazinamide, and ethambutol for two months, followed by isoniazid and rifampicin for four months)—is recommended and has a well-established safety profile during pregnancy. None of these first-line agents has demonstrated clinically significant teratogenic effects when used at therapeutic doses. With appropriate treatment and close monitoring, cure rates of approximately 88% have been reported among pregnant women [31].

Isoniazid readily crosses the placenta but is not associated with teratogenicity. However, the risk of hepatotoxicity is increased during pregnancy, warranting regular monitoring of liver function. Concomitant supplementation with pyridoxine (vitamin B6) is mandatory to prevent peripheral neuropathy in the mother and potential neurological complications in the newborn [31].

Rifampicin is considered safe throughout pregnancy, although its use near term may interfere with vitamin K-dependent coagulation factors. In such cases, prophylactic administration of vitamin K to both the mother and the newborn is recommended. Pyrazinamide is endorsed as safe during pregnancy by the WHO, the International Union Against Tuberculosis and Lung Disease, and most international guidelines, and is routinely included in standard treatment regimens worldwide. Although the CDC historically excluded pyrazinamide due to limited safety data, accumulating evidence now supports its use in pregnancy [20].

Ethambutol is also considered safe and is included in first-line regimens without evidence of teratogenicity, although visual function monitoring is recommended as part of routine care. All treatment should ideally be administered under directly observed therapy to optimize adherence and ensure treatment completion.

For tuberculosis infection, treatment is generally deferred until two to three months postpartum in most cases. However, in women at high risk of progression, such as those with HIV coinfection or recent exposure, treatment should be initiated during pregnancy, with appropriate monitoring [43].

It is important to distinguish the treatment of active tuberculosis disease from tuberculosis preventive therapy during pregnancy, as these interventions differ substantially in their objectives, indications, and therapeutic regimens. Treatment of active tuberculosis is mandatory during pregnancy and aims to achieve maternal cure, interrupt transmission, and reduce the risk of severe maternal, fetal, and neonatal complications associated with untreated disease. In contrast, tuberculosis preventive therapy is intended for pregnant women with tuberculosis infection who are at increased risk of progression to active disease, particularly those living with HIV, recent tuberculosis contacts, or individuals with significant immunosuppression. Preventive therapy does not treat active disease and should only be initiated after exclusion of active tuberculosis through appropriate clinical and diagnostic evaluation.

## 13. Multidrug-Resistant Tuberculosis and Pregnancy

Treatment of multidrug-resistant tuberculosis (MDR-TB) during pregnancy remains challenging and requires individualized multidisciplinary management involving obstetrics, infectious diseases, pulmonology, and, when available, specialized MDR-TB reference centers. Importantly, treatment initiation should not be delayed, as untreated MDR-TB is associated with substantially increased maternal morbidity and mortality, adverse perinatal outcomes, fetal loss, prematurity, and neonatal tuberculosis. Evidence from a systematic review and meta-analysis by Alene et al. [17], including 275 pregnant women with MDR-TB, demonstrated a treatment success rate of 72.2% and favorable pregnancy outcomes in 72.3% of cases, supporting the feasibility of treatment during pregnancy when carefully monitored.

Current WHO recommendations prioritize fully oral MDR-TB regimens and favor the use of Group A agents whenever feasible, including fluoroquinolones, bedaquiline, and linezolid. However, evidence regarding the safety of these agents during pregnancy remains largely based on observational cohorts, pharmacovigilance data, and post-marketing experience, as pregnant women have historically been excluded from most randomized clinical trials involving MDR-TB regimens.

Fluoroquinolones, particularly levofloxacin and moxifloxacin, may be used during pregnancy when clinically indicated. Although early concerns regarding fetal cartilage toxicity originated from animal studies, available human observational data have not consistently demonstrated increased risks of major congenital malformations or severe adverse fetal outcomes. Consequently, WHO guidance allows their use during pregnancy when the expected maternal benefit outweighs theoretical fetal risks.

Bedaquiline-containing regimens now represent a central component of modern MDR-TB treatment strategies and may be considered during pregnancy in selected patients with severe or high-risk disease. Emerging observational evidence suggests that bedaquiline exposure during pregnancy has not been associated with major safety signals or clear increases in adverse pregnancy outcomes. Furthermore, recent evidence from the TB-PRACTECAL trial and subsequent WHO updates has reinforced the growing role of bedaquiline-containing all-oral regimens in MDR-TB management, although pregnancy-specific evidence remains limited because pregnant women were largely excluded from pivotal trials [15]. Nevertheless, when clinically necessary, WHO guidance supports individualized use of bedaquiline-containing regimens during pregnancy with appropriate maternal monitoring, particularly for QT interval prolongation.

In contrast, pretomanid should currently be used with extreme caution during pregnancy. WHO recommendations acknowledge the absence of adequate pregnancy safety data, since pregnant women were excluded from pivotal trials evaluating BPaL- and BPaLM-based regimens. Therefore, pretomanid-containing regimens cannot currently be routinely recommended during pregnancy outside highly individualized expert-based decision-making [15].

Similarly, data regarding delamanid exposure during pregnancy remain limited. Although isolated observational reports and pharmacovigilance data have not identified major teratogenic signals, current evidence is insufficient to establish routine use during pregnancy, and individualized specialist assessment remains necessary before considering its inclusion in MDR-TB regimens [31].

Injectable aminoglycosides should generally be avoided during pregnancy because of the established risk of fetal ototoxicity and nephrotoxicity. Ethionamide and prothionamide are also generally avoided due to concerns regarding teratogenicity, severe gastrointestinal intolerance, and potential exacerbation of pregnancy-related nausea and vomiting.

Overall, treatment selection should be guided by comprehensive drug susceptibility testing, disease severity, prior treatment exposure, maternal clinical condition, and fetal considerations. The main antituberculosis drugs used during pregnancy, together with their indications, safety profiles, and level of supporting evidence, are presented in Table 2. The choice of regimen requires careful balancing of maternal therapeutic benefit against potential fetal risks, recognizing that inadequately treated MDR-TB frequently poses substantially greater danger to both mother and fetus than the potential toxicities associated with appropriately selected second-line drugs. Treatment duration typically ranges from 18 to 24 months, reflecting the complexity of MDR-TB and the need for sustained microbiological control [31].

**Table 2:** Antituberculosis drugs during pregnancy: indications, safety considerations, and strength of evidence.

Drug	Clinical Use	Pregnancy Recommendation	Main Considerations	Strength of Evidence
First-line drugs for drug-susceptible tuberculosis				
Isoniazid	Drug-susceptible TB	Recommended	Risk of hepatotoxicity; pyridoxine supplementation recommended	Strong evidence; WHO guideline-supported
Rifampicin	Drug-susceptible TB	Recommended	CYP450 inducer; potential drug interactions; vitamin K supplementation may be considered near term	Strong evidence; WHO guideline-supported
Pyrazinamide	Drug-susceptible TB	Recommended	Historically limited pregnancy data, but currently considered acceptable and recommended by WHO	Strong evidence/guideline-supported
Ethambutol	Drug-susceptible TB	Recommended	Rare optic toxicity; ophthalmologic monitoring rarely required	Strong evidence/guideline-supported
Second-line drugs for MDR-TB				
Levofloxacin/Moxifloxacin	MDR-TB	May be used when benefits outweigh risks	No consistent evidence of teratogenicity in humans; historical cartilage toxicity concerns derived mainly from animal studies	Limited observational evidence; WHO-supported individualized use
Bedaquiline	MDR-TB	Increasingly used in selected cases	Limited pregnancy-specific data; QT prolongation monitoring recommended	Limited observational evidence; increasing WHO support
Linezolid	MDR-TB	Use with caution	Risk of hematologic toxicity, neuropathy, and gastrointestinal intolerance	Limited observational evidence/expert consensus
Delamanid	MDR-TB	Consider only in highly individualized cases	Insufficient pregnancy-specific safety data	Limited evidence/expert consensus
Pretomanid	MDR-TB	Generally avoided	Pregnant women excluded from pivotal trials; insufficient safety data	Very limited evidence/expert consensus
Aminoglycosides (amikacin, streptomycin, kanamycin)	MDR-TB	Contraindicated/avoid	Risk of fetal ototoxicity and nephrotoxicity	Strong evidence of fetal toxicity
Ethionamide/Prothionamide	MDR-TB	Generally avoided	Possible teratogenicity; severe gastrointestinal intolerance	Limited evidence with safety concerns

Abbreviations: TB, tuberculosis; MDR-TB, multidrug-resistant tuberculosis; WHO, World Health Organization; CYP450, cytochrome P450; QT, QT interval on electrocardiography.

## 14. Tuberculosis and the Newborn

In addition to maternal repercussions, tuberculosis during pregnancy may directly affect the newborn, either through vertical transmission or postnatal exposure, making appropriate neonatal evaluation and management essential.

Neonatal tuberculosis encompasses both congenital infection and disease acquired in the postnatal period, most commonly through aerosol exposure. Congenital tuberculosis may occur through three principal mechanisms: hematogenous transplacental dissemination via the umbilical vein, leading to the formation of a primary hepatic complex; aspiration or ingestion of infected amniotic fluid or vaginal secretions; and, less commonly, direct inoculation during vaginal delivery [20]. The risk is higher in cases of maternal miliary tuberculosis, tuberculous meningitis, genital or pelvic tuberculosis, or placental involvement [35].

Diagnosis of neonatal tuberculosis remains particularly challenging due to its nonspecific clinical presentation, which often overlaps with other neonatal infections. Symptoms typically manifest between the second and third week of life and may include respiratory distress, hepatosplenomegaly, fever, feeding difficulties, and failure to thrive. Chest radiography frequently reveals a miliary pattern, although findings may vary [35].

The diagnosis of congenital tuberculosis is based on the Cantwell criteria, which require the identification of a confirmed tuberculous lesion in the infant in association with at least one additional criterion. These include onset of disease in the first week of life, evidence of a primary hepatic complex or caseating hepatic granulomas, documented tuberculosis of the placenta or maternal genital tract, or exclusion of postnatal transmission [14,32].

Diagnostic evaluation should be comprehensive and may include chest radiography, abdominal ultrasonography, lumbar puncture, and analysis of gastric aspirates, nasopharyngeal aspirates, and stool samples. These specimens should be submitted to smear microscopy for acid-fast bacilli (AFB), molecular testing such as cartridge-based nucleic acid amplification tests (CBNAAT), and mycobacterial culture. When vertical transmission is suspected, placental histopathological examination and mycobacterial culture at delivery are strongly recommended [20].

Treatment of drug-susceptible congenital tuberculosis consists of a combination of isoniazid, rifampicin, pyrazinamide, and ethambutol for a minimum duration of six months, with extended therapy in cases involving the central nervous system or drug resistance [14,31].

Neonatal management should be guided by maternal clinical status at the time of delivery. When the mother is infectious and congenital tuberculosis has been excluded, chemoprophylaxis with isoniazid and rifampicin should be initiated at birth and continued for at least three months, followed by tuberculin skin testing at three months and Bacille Calmette-Guérin (BCG) vaccination if the result is negative. If the mother has completed more than two months of effective treatment before delivery, prophylaxis may be omitted, and only follow-up testing is recommended [35].

Postnatal transmission via aerosols remains the most common route of neonatal infection. A study conducted in South Africa demonstrated a markedly increased incidence of tuberculosis among HIV-infected infants, with a substantial proportion presenting disseminated disease. In cases where the mother is infectious, temporary separation between mother and newborn is recommended to reduce transmission risk, regardless of feeding method [41].

BCG vaccination at birth is recommended in high-burden settings, as it provides significant protection against severe forms of tuberculosis, including miliary disease and tuberculous meningitis. However, it is contraindicated in newborns with suspected or confirmed HIV infection until the immune status has been adequately assessed [31].

## 15. Extrapulmonary Tuberculosis

Extrapulmonary tuberculosis (EPTB) is increasingly recognized during pregnancy, particularly among women coinfected with HIV and those originating from high-burden regions. Epidemiological data from India illustrate this trend, with EPTB accounting for 66.6% of tuberculosis cases during pregnancy in 2019, compared with 20% in 1999 [32].

Diagnosis of EPTB in pregnancy is particularly challenging. Clinical manifestations are often nonspecific and may overlap with physiological changes in pregnancy, including symptoms such as abdominal discomfort, fatigue, and generalized malaise. In addition, reluctance to perform invasive diagnostic procedures during pregnancy may further delay confirmation. A wide spectrum of clinical presentations has been described, including lymphadenitis, pleural tuberculosis, vertebral involvement (Pott disease), pericarditis, meningitis, peritoneal tuberculosis, and genital tuberculosis [38].

Lymph node involvement is the most common presentation and is generally associated with a more favorable prognosis. In contrast, disseminated forms and central nervous system involvement carry a significantly higher risk of morbidity and mortality [32]. Genital tuberculosis is of particular relevance in reproductive health, as hematogenous dissemination to the fallopian tubes, endometrium, ovaries, and cervix may result in tubal infertility, menstrual irregularities, and an increased risk of ectopic pregnancy [20].

Central nervous system involvement, particularly tuberculous meningitis, should be considered in any pregnant woman with risk factors for tuberculosis who presents with persistent headache, neurological symptoms, or altered level of consciousness. Evidence suggests that pregnancies complicated by EPTB are associated with adverse perinatal outcomes, including lower birth weight, increased frequency of small-for-gestational-age infants, and a higher incidence of neonatal respiratory distress syndrome compared with unaffected pregnancies [34].

Diagnostic evaluation should prioritize microbiological confirmation through CBNAAT and mycobacterial culture of appropriate specimens, guided by the site of disease. Chest radiography should be routinely performed to exclude concomitant pulmonary involvement. Treatment follows the same principles as pulmonary tuberculosis, using standard first-line regimens, with extended duration recommended in cases involving the central nervous system [31].

## 16. Postpartum and Breastfeeding

In the postpartum period, issues related to breastfeeding and the risk of transmission must be carefully addressed, balancing the well-established benefits of breastfeeding with measures to prevent neonatal infection.

Large population-based studies have demonstrated an increased incidence of tuberculosis during the postpartum period compared with pregnancy itself [2]. Evidence from large cohort studies indicates that the incidence of tuberculosis is significantly higher in the months following delivery compared with non-gestational periods [18]. This increased risk is thought to reflect rapid immune reconstitution after childbirth, which may unmask previously subclinical infection, in addition to contributing factors such as sleep deprivation, nutritional depletion, and the physiological demands of lactation [38]. The WHO estimates that approximately 49,000 cases of tuberculosis occur annually during the postpartum period [40]. Among women living with HIV, the development of tuberculosis in the postpartum period is associated with a twofold increase in maternal mortality within one year, while their newborns face a threefold higher risk of death [16].

Breastfeeding is not contraindicated in women receiving standard first-line antituberculosis therapy, provided that treatment has been administered for at least two weeks and the mother is no longer considered infectious [20]. First-line drugs are excreted in breast milk at subtherapeutic concentrations, which are insufficient to cause toxicity or to provide effective treatment for the infant. When treatment of the newborn is indicated, a dedicated pediatric regimen with weight-adjusted dosing should be prescribed independently [35].

Supplementation with pyridoxine (vitamin B6) is recommended for all breastfeeding women receiving isoniazid, to prevent neurological complications [20]. In contrast, women with untreated active pulmonary tuberculosis should be temporarily separated from their newborns, regardless of feeding method, as transmission occurs primarily through aerosols and may affect even formula-fed infants [41]. Rifampicin may cause a benign orange discoloration of breast milk, without clinical significance [34].

Breastfeeding is not recommended for women undergoing treatment for multidrug-resistant tuberculosis with regimens that include agents such as bedaquiline, pretomanid, or linezolid, due to the lack of sufficient safety data during lactation [16].

## 17. Contraception

Contraceptive counseling is a fundamental component of care for women of reproductive age undergoing treatment for tuberculosis. Rifampicin, a key drug in first-line regimens, is a potent inducer of cytochrome P450 3A4 (CYP3A4) and P-glycoprotein, significantly accelerating the metabolism of estrogen and progesterone. As a result, the effectiveness of combined oral contraceptives, progestin-only pills, and subdermal implants is markedly reduced [31]. Women receiving rifampicin-containing regimens who rely exclusively on hormonal contraception are therefore at increased risk of unintended pregnancy and should be counseled accordingly.

In contrast, contraceptive methods that act locally, such as copper intrauterine devices and levonorgestrel-releasing intrauterine systems, are not dependent on systemic drug metabolism and thus maintain their efficacy in the presence of rifampicin. For this reason, they are considered among the most reliable contraceptive options for women undergoing antituberculosis treatment. Barrier methods also remain fully effective regardless of concomitant therapy and should be encouraged as part of a comprehensive contraceptive strategy [14].

In women receiving treatment for multidrug-resistant tuberculosis, effective contraception assumes even greater importance. The prolonged duration of therapy and the potential teratogenicity of several second-line drugs necessitate strict avoidance of pregnancy during treatment. In this setting, international guidelines recommend the use of dual protection, typically a highly effective method combined with a barrier method, throughout the treatment period, with postponement of pregnancy until completion of therapy and an additional period of follow-up.

More broadly, comprehensive family planning counseling should be systematically integrated into the care of all women with tuberculosis. This approach should include clear communication regarding the risks associated with pregnancy during treatment, the impact of drug interactions on contraceptive efficacy, and the range of safe and effective contraceptive options available [31].

## 18. Prevention of Multidrug-Resistant Tuberculosis in Pregnant Women

Drug resistance in *Mycobacterium tuberculosis* represents one of the most significant challenges to effective tuberculosis control. It arises from spontaneous chromosomal mutations that confer reduced susceptibility to specific drugs, through mechanisms such as activation of efflux pumps, modification of drug targets, production of drug-inactivating enzymes, and impairment of prodrug activation [44,45,46,47].

MDR-TB is associated with an increased risk of adverse maternal and perinatal outcomes. Nevertheless, pregnancy is not a contraindication to treatment, as untreated disease poses a substantial risk to both mother and fetus. Clinical management should be individualized, taking into account gestational age, disease severity, and drug susceptibility patterns. The risks and benefits of therapy must be carefully balanced, with the primary goal of achieving bacteriological conversion to reduce morbidity and prevent transmission during both pregnancy and the postpartum period [48].

The postpartum period has been increasingly recognized as a phase of heightened susceptibility to tuberculosis activation and clinical deterioration. Epidemiological data indicate that the incidence of active tuberculosis increases by approximately 95% within the first 180 days after delivery. This phenomenon is thought to result from the reversal of pregnancy-associated immunomodulation, particularly the restoration of T helper 1 pro-inflammatory responses, which may lead to clinical unmasking or exacerbation of previously subclinical infection [49,50].

Tuberculosis preventive therapy (TPT) is based on the premise that individuals with tuberculosis infection harbor a low bacillary burden that can be effectively eradicated with one or two drugs, thereby reducing the risk of progression to active disease. The selection of an appropriate TPT regimen depends on multiple factors, including efficacy, availability, safety profile, cost, potential drug interactions, the susceptibility pattern of the index case, and the risk of resistance amplification [51].

Current recommendations from the WHO and the United States Centers for Disease Control and Prevention support the use of levofloxacin for 6–12 months in non-pregnant household contacts of MDR-TB cases. However, there are no established recommendations for MDR-TB preventive therapy during pregnancy, and pregnant women remain excluded from most clinical trials in this field [18,52].

The use of fluoroquinolones during pregnancy is generally avoided unless the expected benefits clearly outweigh potential risks. In cases requiring treatment for MDR-TB, alternative regimens prioritizing safer drugs should be considered, with close fetal monitoring and multidisciplinary management due to the potential teratogenicity of second-line agents [47].

The evidence base supporting TPT in pregnant and postpartum women remains limited and, at times, conflicting, particularly regarding the optimal timing and safety of isoniazid-based regimens. The systematic exclusion of pregnant women from clinical trials has significantly hindered the development of evidence-based guidelines for this population. Notably, no ongoing MDR-TB trials currently include pregnant participants, representing a major barrier to advancing safe and effective therapeutic strategies [47,51,52,53].

In this context, decisions regarding TPT after significant exposure to MDR-TB during pregnancy should be individualized. Clinicians and patients must carefully weigh the potential risks and benefits of available options, and specialist consultation is strongly recommended to guide management [51].

## 19. BCG Vaccination

Vaccination is one of the most cost-effective public health strategies and plays a central role in the WHO End TB Strategy, which aims to eliminate tuberculosis as a public health problem by 2035. Currently, the BCG vaccine remains the only licensed vaccine for tuberculosis prevention. It is a live attenuated vaccine derived from *Mycobacterium bovis*, a pathogen that primarily affects cattle but can also infect humans. Despite its established effectiveness in preventing severe forms of tuberculosis in infants and young children, such as miliary tuberculosis and tuberculous meningitis, BCG shows variable and generally limited efficacy in preventing pulmonary tuberculosis in adolescents and adults and does not reliably prevent primary infection or tuberculosis infection. These limitations underscore the urgent need for more effective vaccines [47,54,55].

According to the WHO recommendations, in high-burden settings, a single dose of BCG should be administered to all newborns as soon as possible after birth. In low-incidence settings, vaccination is targeted to newborns and children at increased risk of exposure, typically based on epidemiological and individual risk factors [46,48].

BCG vaccination is contraindicated during pregnancy because it is a live attenuated vaccine. It should also be avoided in infants with known or suspected immunodeficiency, including those exposed to significant immunosuppressive therapies in utero. In households with suspected or confirmed active tuberculosis, vaccination should be deferred until appropriate evaluation is completed. For infants born to mothers living with HIV, BCG may be administered once the infant is confirmed HIV-negative, typically after early virological testing; recommendations may vary according to local guidelines and feeding practices [46,53].

The risk of tuberculosis infection varies across the life course, with infants being particularly vulnerable, a relative decline during school age, and a subsequent increase during adolescence. This pattern highlights adolescence as a potential window for optimizing vaccination strategies. Emerging evidence suggests that BCG revaccination may enhance immune responses with an acceptable safety profile, although its role in routine immunization programs remains under investigation [56].

## 20. Public Health Perspective

Within the scope of public health, tuberculosis control in pregnant women requires the implementation of integrated strategies that combine effective epidemiological surveillance, timely access to diagnosis and treatment, and educational interventions directed at both the population and healthcare professionals. These measures are essential to reduce transmission and improve maternal and neonatal outcomes.

### 20.1. Monitoring

Monitoring treatment adherence remains one of the greatest challenges in tuberculosis control, as no single method is capable of ensuring consistent adherence. For this reason, healthcare services should adopt a combination of complementary strategies, ideally tailored to individual patient needs [48].

Contact management, particularly in cases of MDR-TB, represents a major challenge for disease elimination. The End TB Strategy has emphasized the importance of preventive therapy, encouraging research to refine clinical practice and improve outcomes [57]. Regardless of the use of TPT, systematic clinical follow-up of exposed individuals, especially during the first 12 months after significant exposure, is essential for early detection and timely treatment of incident disease, thereby reducing morbidity, mortality, and ongoing transmission of *Mycobacterium tuberculosis* [48,51].

The recent introduction of the 6Lfx regimen (six months of daily levofloxacin) as a preventive strategy for MDR-TB contacts represents a significant advance, although important limitations remain. Continued research into alternative drugs and regimens is therefore necessary to support innovation and expand preventive options [55].

In Brazil, Directly Observed Treatment remains a cornerstone of tuberculosis control. This approach requires a committed and humanized relationship between healthcare professionals and patients, combining structured supervision of medication intake, ideally on all working days, with the establishment of trust and therapeutic alliance [48,57].

### 20.2. Education

Community engagement remains insufficiently explored in tuberculosis prevention and control. Social mobilization strategies aim to bring together communities, civil society organizations, and institutional stakeholders to promote awareness, dialogue, and coordinated action. These initiatives involve a wide range of actors, including policymakers, media representatives, non-governmental organizations, healthcare professionals, academic institutions, and patients themselves [51,58].

Educational activities may include community meetings, public campaigns, media engagement, and cultural initiatives. Integration between tuberculosis control programs and immunization services is particularly important, enabling the dissemination of information on prevention strategies, including BCG vaccination, and strengthening training of healthcare professionals [48].

There remains a significant gap in knowledge among pregnant women regarding tuberculosis, its complications, and its impact on maternal and neonatal health. Educational interventions should emphasize the importance of treatment adherence, the risks associated with delayed or interrupted therapy, and the benefits of timely management. Counseling sessions, combined with psychological support, may improve adherence and increase confidence in achieving favorable pregnancy outcomes. All pregnant women receiving antenatal care should be informed that the benefits of tuberculosis treatment outweigh the potential risks of pharmacological therapy [42,52].

More broadly, population-level education is essential to reinforce that tuberculosis, although highly transmissible, is a treatable and curable disease when appropriate diagnostic and therapeutic support is available [36]. Vaccination opportunities should also be leveraged to provide health education and to monitor BCG coverage, supporting strategies aimed at maintaining tuberculosis control targets [42,48].

### 20.3. National Policy

The increasing complexity of drug-resistant tuberculosis demands equally robust public health responses, supported by sustained policies, expanded laboratory capacity, and rational use of newer therapeutic agents. The future trajectory of MDR-TB control in Brazil will depend on effective integration of technological innovation, structured surveillance systems, and patient-centered care, enabling early identification and containment of resistant strains before they become widespread [57].

Globally, tuberculosis control is aligned with the United Nations Sustainable Development Goals, which include ending the tuberculosis epidemic by 2030 (Goal 3). To support this objective, WHO Member States adopted the End TB Strategy in 2015, aiming to reduce tuberculosis-related deaths by 95% by 2035 compared with 2015 levels. In Brazil, implementation of this strategy is coordinated across multiple levels of the Unified Health System (SUS), including healthcare networks, intergovernmental commissions, and policy coordination spaces that foster political commitment and social mobilization [48,58].

The End TB Strategy is structured around three core pillars: (1) integrated, patient-centered care and prevention, focusing on early diagnosis, treatment, and vaccination; (2) supportive policies and systems that strengthen health and social sectors; and (3) intensified research and innovation to advance new tools and strategies for tuberculosis control [54,57].

Effective implementation of these measures requires coordinated action among federal, state, and municipal authorities, ensuring alignment of responsibilities and sustained commitment to tuberculosis control.

## 21. Vaccine Research

The immune response to MTB infection involves a complex and not yet fully elucidated network of cellular interactions, which continues to pose challenges for the development of effective vaccines. Protection against severe and disseminated forms of tuberculosis, particularly in children, has been associated with a T-helper 1 (Th1)–type immune response mediated by CD4+ T lymphocytes producing interferon gamma (IFN-γ). Despite this response, the immune system is often unable to achieve complete eradication of the bacillus, resulting in persistence of tuberculosis infection [54].

Advances in tuberculosis vaccinology have led to the development of multiple vaccine candidates, currently at various stages of clinical and preclinical evaluation. These strategies aim either to replace the BCG vaccine or to enhance its protective efficacy through prime-boost approaches. A critical step in this process is antigen selection, which involves identifying components capable of eliciting robust and protective immune responses. In this context, advances in bioinformatics and artificial intelligence have substantially improved the identification and characterization of candidate antigens [55].

These technologies enable the prediction of antigen immunogenicity and antigenicity, the modeling of three-dimensional protein structures, and the simulation of molecular interactions through docking analyses. Artificial intelligence has also accelerated high-throughput screening of candidate antigens and facilitated the integration of complex biological datasets. Moreover, it has opened new avenues for personalized vaccine development by incorporating individual genetic and immunological profiles into antigen selection and vaccine design [55].

Among the different platforms under investigation, live attenuated vaccines have emerged as promising candidates due to their ability to mimic natural infection and induce strong cellular immune responses. Examples include MTBΔsigH, an attenuated strain with deletion of the sigH gene, and MTBΔlpqS, a mutant strain capable of enhancing IFN-γ production while reducing anti-inflammatory cytokines such as interleukin-10. In addition, *Mycobacterium paragordonae*, a temperature-sensitive mycobacterium, has demonstrated the capacity to induce dendritic cell maturation and Th1 responses in experimental models, with associated reductions in bacterial load and pulmonary inflammation [55].

Viral vector-based vaccines have also gained prominence, given their efficiency in delivering antigens and eliciting potent immune responses. Candidates such as LV::li-HAEPA and SeV85AB encode MTB antigens and promote targeted immune activation [55].

Other innovative approaches include recombinant BCG vaccines, DNA-based platforms such as DNA-hsp65, and emerging mRNA vaccines, all of which expand the range of possibilities in tuberculosis vaccine development. Together, these strategies encompass a spectrum ranging from traditional whole-cell formulations to highly engineered molecular platforms, reflecting the intensity of current research efforts and the urgent need for more effective tools to control tuberculosis globally [55].

## 22. Evidence Gaps

Significant gaps persist in the diagnosis and management of tuberculosis, contributing to suboptimal outcomes in disease control and prevention and highlighting critical areas for policy development. These gaps occur across multiple levels, including the patient, family, community, and healthcare system, as well as within the interactions between patients and healthcare providers. Strengthening patient-centered diagnostic networks and addressing deficiencies across the entire tuberculosis care cascade are essential to achieving global eradication targets [16,59].

Despite existing strategies, further innovations in diagnostic technologies, therapeutic regimens, and expansion of tuberculosis care services remain necessary to meet the goals of the WHO End TB Strategy, which aims to reduce tuberculosis mortality by 95% by 2035 [16,59]. A substantial proportion of patients remain undiagnosed or experience significant diagnostic delays, underscoring the need to close the gap between disease incidence and case notification, increase rates of bacteriological confirmation, and ensure early initiation of effective treatment [59].

In the field of prevention, approximately one-fifth of identified research gaps relate to clinical evidence supporting large-scale implementation of tuberculosis preventive therapy (TPT). These include the need for improved diagnostic tools, validated biomarkers of disease progression, and more effective treatment strategies for contacts of drug-resistant tuberculosis cases. Additional gaps concern optimal technologies for testing, treatment, and infection control, as well as the development of biomarkers to monitor therapeutic response and predict adverse outcomes [16].

Research plays a central role in informing clinical guidelines and public health policies. As emphasized in the Global TB Research and Innovation Strategy, research is not only a scientific endeavor but also a political commitment, requiring sustained investment, effective implementation, and broad dissemination guided by principles of accessibility, efficiency, and equity. Achieving the goal of tuberculosis elimination by 2030 will require a substantial increase in global investment in research [16].

In pregnant women, important gaps remain, particularly in the collection of data on maternal, fetal, and neonatal outcomes, including long-term postpartum follow-up. There is also a critical need for pharmacokinetic and safety studies to determine optimal dosing of key drugs, especially newer agents such as levofloxacin and bedaquiline [16].

Future research priorities should include neonatal pharmacokinetic studies and population-based modeling to better understand transplacental drug transfer and drug excretion in breast milk [44]. The establishment of global registries to systematically collect data on efficacy, safety, tolerability, and maternal–fetal outcomes of multidrug-resistant tuberculosis regimens is urgently needed. Recent international consensus supports the inclusion of pregnant and lactating women in phase III clinical trials for MDR-TB, unless there are compelling reasons for exclusion [60].

The identification of reliable biomarkers capable of predicting progression from tuberculosis infection to active disease remains an important unmet need, particularly in pregnant populations, where immunological adaptations may influence disease expression. More robust immunological studies are therefore required to elucidate these mechanisms [18].

Finally, the development of new vaccines represents a major priority, to achieve broader and more durable protection, including against drug-resistant strains and across diverse populations. Combination strategies integrating vaccination and therapeutic approaches may further enhance effectiveness and contribute to overcoming current limitations in tuberculosis control [54,55].

## 23. Conclusions

Tuberculosis during pregnancy remains a persistent clinical and public health challenge, with profound implications for both maternal and neonatal outcomes. Its burden is magnified by diagnostic delays, therapeutic complexities, and systemic inequities in access to care. Early identification and timely treatment, supported by vigilant clinical follow-up, are essential to mitigate these risks. However, the effective control of tuberculosis in pregnancy extends beyond the clinical setting. It demands integrated public health responses, encompassing surveillance, education, and equitable access to care, capable of addressing both biological vulnerability and social determinants of disease. Ultimately, progress in this field will depend on sustained investment in research, particularly in populations historically underrepresented in clinical studies. Advancing knowledge in diagnostics, therapeutics, and prevention will not only improve outcomes for pregnant women and their children but also represent a critical step toward the broader goal of tuberculosis elimination.

## Figures and Tables

**Table 1:** Practical diagnostic modalities for tuberculosis during pregnancy: sample types, clinical indications, diagnostic performance, pregnancy-specific limitations, and main advantages.

Diagnostic Modality	Sample Type	Practical Indication	Approximate Diagnostic Performance	Pregnancy-Specific Limitations	Main Advantages
Symptom screening	Clinical evaluation	Initial screening at every antenatal visit, especially in high-burden settings or high-risk patients (HIV, close contact, immunosuppression)	High sensitivity for screening; low specificity	Symptoms such as fatigue, dyspnea, night sweats, and mild anemia may overlap with physiological pregnancy changes	Simple, inexpensive, universally applicable
TST	Intradermal PPD reaction	Detection of tuberculosis infection, particularly in contacts or high-risk populations	Sensitivity: ~70–80%; specificity variable depending on BCG vaccination and environmental mycobacteria exposure	Cannot distinguish infection from active disease; false-negative results may occur in immunosuppression or advanced disease	Safe during pregnancy; widely available
IGRAs	Peripheral blood	Detection of tuberculosis infection, especially in BCG-vaccinated populations	Specificity generally >95%; sensitivity ~75–85%	Pregnancy-associated immunological changes may attenuate interferon gamma responses; limited availability and higher cost	Higher specificity than TST; unaffected by prior BCG vaccination
IP-10-based assays (emerging biomarker)	Peripheral blood	Investigational adjunctive biomarker for tuberculosis infection during pregnancy	Preliminary studies suggest preserved diagnostic performance despite altered interferon gamma responses	Not yet standardized or routinely available; limited pregnancy-specific validation	Potentially more robust biomarker during pregnancy
Smear microscopy	Sputum	Initial microbiological evaluation of suspected pulmonary tuberculosis	Sensitivity: ~40–60%; specificity high in endemic settings	Reduced sensitivity in paucibacillary disease and HIV coinfection	Rapid, inexpensive, widely available
GeneXpert MTB/RIF	Sputum (or other clinical specimens)	First-line molecular diagnostic test for suspected tuberculosis and rifampicin resistance detection	Sensitivity > 80%; specificity ~98–99%	May be negative in paucibacillary disease	Rapid diagnosis with simultaneous rifampicin resistance detection
Xpert MTB/RIF Ultra	Sputum (or extrapulmonary specimens)	Improved molecular detection in paucibacillary disease, HIV coinfection, or smear-negative tuberculosis	Higher sensitivity than standard [20] Xpert MTB/RIF, especially in paucibacillary disease	Slightly reduced specificity in some settings due to trace-positive results	Improved sensitivity for early or low-bacillary-burden disease
Mycobacterial culture	Sputum, bronchoalveolar lavage, tissue, placental or extrapulmonary specimens	Confirmatory diagnosis and drug susceptibility testing	Gold standard [20]; highest sensitivity	Long turnaround time (weeks) may delay definitive confirmation	Essential for drug susceptibility testing and resistant tuberculosis evaluation
LF-LAM assay	Urine	Rapid diagnostic adjunct in HIV-positive pregnant women with advanced immunosuppression	Highest yield in patients with low CD4 counts	Limited utility in immunocompetent patients	Rapid bedside testing; useful in severe HIV-associated disease
Chest radiography	Thoracic imaging	Initial imaging evaluation of suspected pulmonary tuberculosis	Moderate sensitivity; lower sensitivity for early or miliary disease	Imaging findings may be atypical or normal, especially in HIV coinfection; concerns regarding radiation exposure may delay testing	Safe with abdominal shielding; widely available
Chest CT	Thoracic imaging	Selected cases: suspected miliary disease, inconclusive chest radiography, severe clinical deterioration, immunocompromised patients, complex thoracic involvement	Higher sensitivity than chest radiography for subtle, early, atypical, or disseminated disease	Higher maternal radiation exposure; requires individualized risk–benefit assessment	Superior anatomical characterization and detection of early parenchymal abnormalities
Histopathological placental evaluation	Placental tissue	Suspected congenital or placental tuberculosis	Variable sensitivity; may support diagnosis when combined with microbiology	Paucibacillary disease may limit bacilli detection	May demonstrate granulomatous inflammation, necrosis, and placental involvement

Abbreviations: HIV, human immunodeficiency virus; TST, tuberculin skin test; PPD, purified protein derivative; IGRA, interferon gamma release assay; IP-10, interferon gamma-induced protein 10; GeneXpert MTB/RIF, automated nucleic acid amplification assay for detection of Mycobacterium tuberculosis and rifampicin resistance; LF-LAM, lateral flow urine lipoarabinomannan; CT, computed tomography; BCG, Bacille Calmette-Guérin; CD4, cluster of differentiation 4.

## Data Availability

The data presented in this study are available on request from the corresponding author.
